# Editorial: Insights in neurotrauma: 2021

**DOI:** 10.3389/fneur.2023.1094723

**Published:** 2023-01-24

**Authors:** Mårten Risling

**Affiliations:** Department of Neuroscience, Karolinska Institutet, Solna, Sweden

**Keywords:** traumatic brain injury (TBI), trauma, nervous system, clinical, experimental - animal models

Insights collections is an initiative from Frontiers to highlight the recent advances in research within different fields of science. With this background, it was decided to invite authors to submit articles that could illustrate the advancements in understanding, diagnosis, or treatment of neurotrauma. The collection is titled “*Insights in Neurotrauma 2021*.” The ten published articles cover several aspects of neurotrauma, in both experimental studies and clinical articles. The authors were encouraged to identify the greatest challenges in their sub-disciplines, and how to address such challenges.

The first published article in the collection is “*Age-At-Injury Influences the Glial Response to Traumatic Brain Injury in the Cortex of Male Juvenile Rats*” by Green et al.. This is an experimental study on juvenile rats. It was hypothesized that rats injured at post-natal day (PND) 17 would exhibit a greater glial response, that would persist into early adulthood, compared to rats injured at PND35. It is concluded that TBI at an early age may trigger a more prominent glial response. This article adds to the understanding of how age affects the response to neurotrauma and that the juvenile stage can show dynamic differences in a matter of days in terms of glial response.

The second article is “*Analgesia in the Neurosurgical Intensive Care Unit*,” by Kvolik et al.. This review article describes important considerations for pain control of neurosurgical intensive care patients. The preservation of adequate cerebral perfusion and oxygenation while managing intracranial pressure, mechanical ventilation, stability of circulation and fluid balance, temperature and glycemic control in neurosurgical patients is highly complex, and adequate pain control can improve outcomes. The article discusses pain control after a craniotomy. The review also describes the treatment of paroxysmal sympathetic hyperactivity, respiratory depression, gastroparesis, bowel paralysis, and hypotension. The treatment of addicted patients is another aspect covered in this review. Among unsolved questions, the article discusses whether the choice of analgesics may influence neurological recovery.

The article “*The Glial Cells Respond to Spinal Cord Injury*” by Wang et al. is a review of the response of astrocytes, oligodendrocytes, and microglia after spinal cord injury. The role of the different glial cells in the normal central nervous system (CNS) and the change after trauma is reviewed and discussed in this paper. The molecular pathways involved in the transition of the different glial cells function after injury are reviewed and discussed.

“*Decline in the Incidence of Chronic Subdural Hematoma During the Coronavirus Disease 2019 Pandemic: A Retrospective Single-Center Descriptive Study*” by Maeoka et al. is an article describing the change in the incidence of chronic subdural hematoma during the COVID-19 pandemic in Akashi city, Japan. The authors identify significant associations between the COVID-19 pandemic and a decline in the number of head traumas and chronic subdural hematomas.

The article ”*Research Hotspots and Trends of Peripheral Nerve Injuries Based on Web of Science From 2017 to 2021: A Bibliometric Analysis*” by Zhang et al. is a systematic review of articles and reviews on peripheral nerve injury from 2017 to 2021, extracted from the Web of Science. The dorsal root ganglion and satellite glial cells are identified as hot topics in areas such as neuropathic pain relief. Tissue engineering techniques and the repair of Schwann cell phenotype were also discussed in the context of focus of future research.

”*Evaluation of decompressive craniectomy in mice after severe traumatic brain injury*” by Liu et al. is an article that describes the development of an experimental model for treatment of severe traumatic brain injury (TBI) in mice with decompressive craniectomy. The results show that the group that had been treated with decompressive craniectomy had significantly lower intracranial pressure and better neurological and motor function at 24 h after injury. However, at later stages, it is found that decompressive craniectomy had a negative effect on neurological function.

“*A Novel Therapeutic Approach With Sodium Pyruvate on Vital Signs, Acid–Base, and Metabolic Disturbances in Rats With a Combined Blast and Hemorrhagic Shock*” by Saha et al.. This study shows that sodium pyruvate resuscitation significantly improves the mean arterial pressure, heart rate, pulse pressure, hemodynamic stability, and autonomic response after hemorrhagic shock and/or blast injury.

“*Opinion: The Potential Role of Amyloid Beta Peptides as Biomarkers of Subconcussion and Concussion*” by Boutté et al.. In this article, the authors discuss the potential value of A-beta as a biomarker for subconcussion and concussion on the basis of findings in a limited cohort and previous data.

”*Immunoexpression of MMP-8 and MMP-9 in chronic subdural hematoma*” by Su et al.. In this study, the authors analyze matrix metallopeptidase (MMP)-8 and MMP-9 in 83 patients with chronic subdural hematoma and 50 normal individuals. The concentration of MMP-8 is significantly higher in peripheral blood from the normal group than that in the chronic subdural hematoma cases, whereas the level of MMP-9 is lower in the normal group.

The final paper published in this collection is “*Serum vitamin E level and functional prognosis after traumatic brain injury with intracranial injury: A multicenter prospective study*” by Park et al.. This study aims to evaluate the prognostic value of vitamin E on functional outcomes of traumatic brain injury (TBI) patients and is performed as a multi-center prospective cohort study. The data indicate that low serum vitamin E level is associated with poor prognosis at 1 and 6 months after TBI.

In summary, the Insights in Neurotrauma article collection represents an interesting mixture of ongoing research in neurotrauma, including experimental and clinical studies. Traumatic injuries to the nervous system represent an extensive field of various clinical problems. Many of the current problems are represented in this article collection. This is not a complete overview of the current research but can be regarded as a snapshot of different lines of research.

## Author contributions

The author confirms being the sole contributor of this work and has approved it for publication.

